# Impact of a smartphone-connected remote monitoring system on self-management continuity and health awareness in cardiovascular outpatients: an exploratory survey

**DOI:** 10.1093/ehjdh/ztae101

**Published:** 2024-12-24

**Authors:** Masanobu Ishii, Masahiro Yamamoto, Yasuhiro Otsuka, So Ikebe, Yoshinori Yamanouchi, Kenichi Tsujita, Taishi Nakamura

**Affiliations:** Department of Cardiovascular Medicine, Graduate School of Medical Sciences, Kumamoto University, 1-1-1 Honjo, Chuo-ku, Kumamoto, Kumamoto 860-8556, Japan; Department of Medical Information Science, Graduate School of Medical Sciences, Kumamoto University, 1-1-1 Honjo, Chuo-ku, Kumamoto, Kumamoto 860-8556, Japan; Department of Cardiovascular Medicine, Graduate School of Medical Sciences, Kumamoto University, 1-1-1 Honjo, Chuo-ku, Kumamoto, Kumamoto 860-8556, Japan; Department of Cardiovascular Medicine, Graduate School of Medical Sciences, Kumamoto University, 1-1-1 Honjo, Chuo-ku, Kumamoto, Kumamoto 860-8556, Japan; Department of Cardiovascular Medicine, Graduate School of Medical Sciences, Kumamoto University, 1-1-1 Honjo, Chuo-ku, Kumamoto, Kumamoto 860-8556, Japan; Department of Clinical Investigation, Kumamoto University Hospital, 1-1-1 Honjo, Chuo-ku, Kumamoto 860-8556, Japan; Department of Cardiovascular Medicine, Graduate School of Medical Sciences, Kumamoto University, 1-1-1 Honjo, Chuo-ku, Kumamoto, Kumamoto 860-8556, Japan; Department of Clinical Investigation, Kumamoto University Hospital, 1-1-1 Honjo, Chuo-ku, Kumamoto 860-8556, Japan; Department of Medical Information Science, Graduate School of Medical Sciences, Kumamoto University, 1-1-1 Honjo, Chuo-ku, Kumamoto, Kumamoto 860-8556, Japan; Department of Clinical Investigation, Kumamoto University Hospital, 1-1-1 Honjo, Chuo-ku, Kumamoto 860-8556, Japan

**Keywords:** mHealth, Smartphone-connected remote monitoring system, Self-management

## Abstract

**Aims:**

Cardiovascular diseases are a leading cause of death globally, and effective self-management is critical for patient outcomes. Integrating Internet of Things-enabled devices with smartphone applications presents a novel approach to enhancing self-management, yet challenges with digital literacy and device usability persist, especially among the elderly. This study aimed to evaluate the adherence, ease of use, and impact on health awareness of a smartphone-connected remote monitoring system among cardiovascular outpatients in Japan.

**Methods and results:**

We conducted a single-centre, prospective survey at Kumamoto University Hospital involving 10 cardiovascular outpatients (median age: 72.5 years) including heart failure (*n* = 2), hypertension (*n* = 3), post-cardiac surgery (*n* = 2), and others (*n* = 3). Participants received Bluetooth-enabled monitoring devices and a smartphone app for automatic data synchronization. Adherence, ease of use, and changes in health awareness were assessed through a structured questionnaire. The study found that 8 of 10 participants adhered to daily monitoring, with an average usage period of 48 days. Nine of 10 required minimal support with device use and 8 of 10 reported increase in health awareness. Seven of 10 indicated they could continue using it long term. The average recommendation score was 8.8/10. The timely detection of asymptomatic paroxysmal atrial fibrillation in one patient highlighted the system's potential clinical benefits.

**Conclusion:**

This pilot study suggests that a smartphone-connected remote monitoring system may enhance self-management practices and health awareness among cardiovascular outpatients. While the findings are promising, larger studies with longer follow-up periods are needed to confirm these results and evaluate the system's impact on clinical outcomes.

## Graphical Abstract

**Graphical Abstract:**
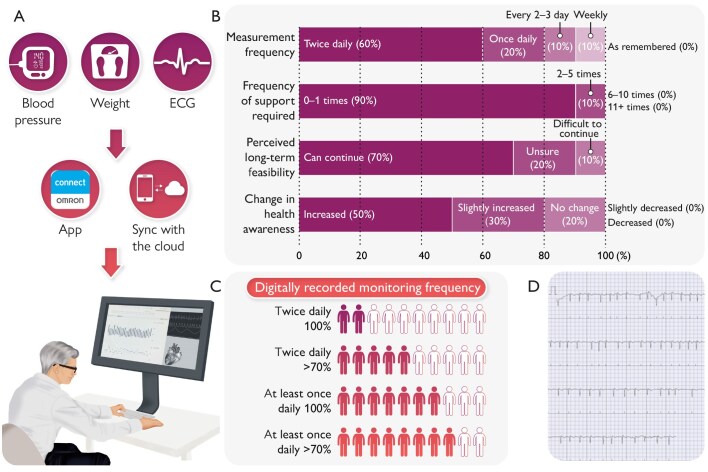

## Introduction

Cardiovascular diseases remain a leading cause of morbidity and mortality worldwide, with Japan facing particularly high prevalence among its super-aged population. Effective self-management, including regular monitoring of vital signs, is crucial for these patients. Traditional methods often encounter poor adherence and inconsistent data recording. The Internet of Things and smartphone applications offer new possibilities for enhancing self-management. Several studies have shown that digital health interventions can improve clinical outcomes for cardiovascular diseases by supporting medication adherence and lifestyle changes.^1–5^ However, older cardiovascular patients may need additional support in using these digital tools.^6^ Training both healthcare providers and patients in remote monitoring systems has been recommended to improve digital literacy and mobile health (mHealth) adoption.^5^ Few studies have investigated systems that automatically transfer patient data to healthcare providers via apps and server, which may support sustained self-monitoring and healthcare interventions with minimal training, potentially improving adherence and data accuracy. To optimize digital health implementation in a super-aged society, further research is needed on the digital literacy and accessibility of devices among older adults. This study aimed to evaluate the usability, continuity of self-management, and impact of a smartphone-connected remote monitoring system on health awareness among a broad age range of cardiovascular outpatients in Japan.

## Methods

**Figure 1:**
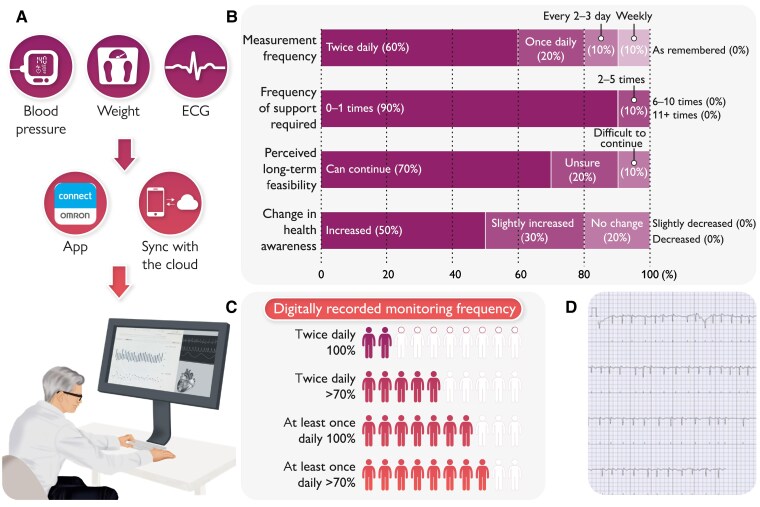
Results of patient survey after using a smartphone-connected remote monitoring system. (*A*) Internet of Things measurement device and a smartphone app with automatic synchronization to a cloud system allow healthcare professionals to check patients’ vital data. The system interface enables healthcare professionals to view patients’ measurement data over time. (*B*) This system demonstrates that the majority of vital measurements are performed once or twice daily, with little need for support from others, indicating high continuity in system usage in self-assessment questionnaire. (*C*) Proportion of patients achieving digitally recorded monitoring frequency during the follow-up period. (*D*) The mobile electrocardiogram device detected asymptomatic paroxysmal atrial fibrillation in one patient.

This single-centre, prospective, exploratory survey used a structured, multiple choice questionnaire to collect data from 10 outpatients with cardiovascular diseases at Kumamoto University Hospital. Outpatients with various cardiovascular conditions were included to assess the feasibility and impact of self-monitoring across a broader spectrum of cardiovascular conditions, including heart failure. Unlike traditional self-management methods relying on non-connected devices and manual data recording or in-person monitoring, this study implemented a smartphone-connected system to enable automatic data transfer and remote monitoring. Participants received a Bluetooth-enabled upper arm blood pressure (BP) monitor (HCR-7501T, OMRON Healthcare Co, Ltd), weighing scale (HN-300T2, OMRON Healthcare Co, Ltd), and/or portable electrocardiograph (HCG-8060T, OMRON Healthcare Co, Ltd) (*Figure 1A*). The participants used these devices and OMRON connect app (OMRON Healthcare Co, Ltd) for automatic data syncing from March to June 2024. They were instructed to measure BP and pulse rate twice daily and weight once daily; electrocardiograms were recorded at their discretion, such as during palpitations or other symptoms. The measured data were automatically transmitted via the connected devices. The app with automatic synchronization to a cloud system allows healthcare professionals to check patients’ vital data (*Figure 1A*). The healthcare professionals monitored participants’ data approximately three times per week, primarily to confirm that participants were consistently performing the required measurements and that the data were being accurately recorded in the system. At their next outpatient visit, participants completed a questionnaire evaluating their experience with the devices and app. The questionnaire consisted of items with three to five response options for each question, from which respondents were asked to select one option. It assessed measurement frequency, ease of use (frequency of support required), perceived long-term feasibility, changes in health awareness, and likelihood of recommending the system (rate on a scale of 10 points). All procedures were conducted in accordance with the Declaration of Helsinki and its amendments. This study received approval from the Human Ethics Review Committee of Kumamoto University Hospital, Japan (Rinri No. 2908). Written informed consent was obtained from each participant.

## Results

A total of 10 outpatients from the cardiovascular department participated in this study, with an average usage period of 48 days. The median age of the participants was 72.5 years (interquartile range: 52.75–76.75), and 90% were male. The participants included patients with a range of primary cardiovascular diagnoses: heart failure (two patients), hypertension (three patients), ischaemic heart disease (one patient), aortic disease (one patient), history of ventricular fibrillation (one patient), and post-cardiac surgery for valvular or congenital heart disease (two patients). The majority of participants (6 of 10 participants) reported measuring their vital signs twice daily. Two patients measured once daily, while one patient measured every 2–3 days, and another measured weekly (*Figure 1B*). Digitally recorded data showed that while adherence varied, most participants maintained monitoring on over 70% of days, although only a few achieved twice-daily measurements without any missed days (*Figure 1C*). As shown in *Figure 1B*, most participants (9 of 10) required minimal support (0–1 instances) in using the devices and app. Only one patient needed moderate support (2–5 instances). Five participants reported an increase in health awareness, while three noted a slight increase, and two perceived no change in their health awareness. The majority of participants (7 of 10) believed they could continue using the system in the long term. Two patients were unsure about their ability to continue, and one patient found it difficult to continue. On a 10-point scale assessing the likelihood of recommending the system, most participants (6 of 10) gave the highest rating of 10 points. Two patients rated it 9 points, while one patient each rated it 5 and 3 points, respectively. The average recommendation score was 8.8 out of 10. A key finding was that the mobile electrocardiogram device used in this study detected asymptomatic paroxysmal atrial fibrillation in one patient (*Figure 1D*), leading to timely medical intervention. One of the main issues reported was difficulty with automatic data transfer from the devices to the app. Some participants noted that data from the BP monitor occasionally took several minutes to appear in the app after measurement. This delay was possibly due to Bluetooth connectivity issues, as the distance between devices was not a factor, and no similar issues were observed with the weight scale or portable electrocardiogram device.

## Discussion

Our findings suggest that the smartphone-connected remote monitoring system may have a positive impact on self-management practices and health awareness among cardiovascular outpatients. The high measurement frequency (8 of 10 participants measuring at least daily) indicates good adherence to the monitoring regimen. This consistent data collection could provide physicians with more comprehensive and reliable information for patient management. The system's ease of use, with most patients (9 of 10) requiring minimal support, suggests that it could be widely adopted without significant technical barriers. This is crucial for the successful implementation of any health technology, particularly among older patients who may be less familiar with digital devices. The observed improvement in health awareness (8 of 10 reporting some increase) is a promising outcome. Enhanced awareness could potentially lead to better self-management practices and improved health outcomes. The high long-term feasibility (7 of 10 believing they could continue using the system) and the strong likelihood of recommending the system (average score of 8.8/10) indicate a positive perception of its value among participants.

It is important to acknowledge the challenges that mHealth interventions can cause, particularly for older adults who comprise a significant portion of cardiovascular patients.^6^ Age-associated changes in cognitive, physical, and sensory abilities can impact the usability of mHealth applications.^6^ However, addressing these issues requires avoiding the perpetuation of ageist stereotypes. A proactive, supportive approach that fosters skill development and builds digital health literacy among older patients can help bridge these gaps. By providing accessible training and resources, older adults can be empowered to use digital health tools effectively, potentially enhancing their self-management practices and health outcomes. Furthermore, attention to inclusive design principles in developing digital health products can improve usability for older users. Prioritizing intuitive interfaces, clear visual and auditory cues, and adaptability for diverse needs will support the broader adoption of these tools among older adults. Despite these potential obstacles, our study's high rates of adherence and user positive perception of its value suggest that carefully designed mHealth interventions can overcome many of these issues. In selecting suitable candidates for digital health interventions, it is crucial to move beyond age as the primary criterion. Instead, a personalized approach that considers each patient’s interest, preferences, and digital skills is recommended, as many older adults were highly capable and engaged with technology in this study. This individualized selection process, alongside thoughtfully designed mHealth interventions, can support a more inclusive and effective use of digital health tools, allowing patients of all ages to engage confidently with these innovations.

However, this study has several limitations. Firstly, the small sample size limits the generalizability of the results. Secondly, the short duration of the study could not allow for the assessment of long-term adherence or health outcomes. Thirdly, given the sample size of 10 participants, monitoring by a single healthcare professional was sufficient. For future large-scale implementation, additional consideration would be needed regarding optimal monitoring frequency and the allocation of healthcare resources to ensure effective and sustainable monitoring.

In conclusion, this pilot study demonstrated that a smartphone-connected remote monitoring system can enhance health awareness among cardiovascular outpatients, which may, in turn, support future improvements in self-management continuity. The system demonstrated ease of use, was well received by participants, and showed potential for promoting sustained self-management practices. Larger, long-term studies are needed to confirm these findings and assess the impact on clinical outcomes.

## Data Availability

The data underlying this article will be shared on reasonable request to the corresponding author.
